# Increased Expression of Extracellular Vesicles Is Associated With the Procoagulant State in Patients With Established Rheumatoid Arthritis

**DOI:** 10.3389/fimmu.2021.718845

**Published:** 2021-07-29

**Authors:** Aleksandra Stojanovic, Mirjana Veselinovic, Yanan Zong, Vladimir Jakovljevic, Iva Pruner, Aleksandra Antovic

**Affiliations:** ^1^Department of Pharmacy, Faculty of Medical Sciences, University of Kragujevac, Kragujevac, Serbia; ^2^Department of Internal Medicine, Faculty of Medical Sciences, University of Kragujevac, Kragujevac, Serbia; ^3^Department of Molecular Medicine & Surgery, Karolinska Institutet, Stockholm, Sweden; ^4^Department of Physiology, Faculty of Medical Sciences, University of Kragujevac, Kragujevac, Serbia; ^5^Department of Human Pathology, I.M. Sechenov First Moscow State Medical University, Moscow, Russia; ^6^Department of Medicine, Division of Rheumatology, Karolinska Institutet, Stockholm, Sweden; ^7^Academic Specialist Center, Center for Rheumatology, Stockholm Health Services, Stockholm, Sweden

**Keywords:** rheumatoid arthritis, extracellular vesicles, hemostasis, inflammation, fibrin structure

## Abstract

This study sought to identify different subpopulations of extracellular vesicles (EVs) in plasma from female patients with established rheumatoid arthritis (RA) in relation to the activation of coagulation and fibrin formation in these patients. Forty women were included in the study, 20 patients and 20 age-matched healthy controls. The mean disease duration in patients was 13.0 (5.0–25.0) years, with medium to high disease activity despite ongoing treatment with low-dose prednisolone and methotrexate. There were no differences between the investigated groups regarding the presence of traditional cardiovascular risk factors. The concentration of phosphatidylserine-positive (PS^+^) EVs; platelet (CD42a^+^), leucocyte (CD45^+^), monocyte (CD14^+^), and endothelial (CD144^+^)-derived EVs; and EVs-expressing tissue factor (CD142^+^), P-selectin (CD62P^+^), and E-selectin (CD62E^+^) were determined by flow cytometry analysis. Overall hemostasis potential (OHP) was assessed to follow the hemostatic disturbances, including the parameters for overall coagulation potential (OCP) and overall fibrinolytic potential (OFP). Fibrin clot turbidity was measured together with clot lysis time, and scanning electron microscopy was performed. Increased concentrations of PS^+^, CD42a^+^, CD142^+^, CD45^+^, CD14^+^, and CD62P^+^ EVs were found in plasma from patients with RA compared to healthy controls, and the concentrations of PS^+^, CD42a^+^, CD14^+^, and CD62P^+^ EVs were positively correlated with the inflammatory parameters in RA patients. Positive correlations were also found between the levels of PS^+^ and CD42a^+^ EVs and OCP as well as between the levels of PS^+^, CD42a^+^, and CD62P^+^EVs and OHP. The levels of PS^+^, CD42a^+^, CD14^+^, CD62P^+^, and CD62E^+^ EVs were negatively correlated with OFP. Elevated levels of circulating EVs of different cell origins were found in patients with established RA, in relation to the inflammatory burden and coagulation activation in the disease.

## Introduction

Rheumatoid arthritis (RA) is a chronic, inflammatory, autoimmune disease causing synovitis and destructive arthritis and is accompanied by out-of-joint disease manifestations, a strong systemic inflammatory response with accelerated development of atherosclerosis and shortened life expectancy and as such represents a significant burden for both individuals and society (1–5). The most common onset of illness is in the forties (6), with a significantly higher disease activity in the female population (7). Patients with RA have high prevalence of cardiovascular disease (CVD) as well as an increased risk of developing fatal cardiovascular events such as acute myocardial infarction, stroke, and heart failure (8, 9).

The pathogenesis of RA is complex and comprises interactions between genetics, epigenetic modifications, and environmental factors such as smoking, all of which contribute to the production of specific autoantibodies against citrullinated proteins (ACPAs), systemic inflammatory response, and joint destruction.

Recently, the role of microparticles was implicated in the pathogenesis of RA. Microparticles, also called extracellular vesicles (EVs), are small membrane-coated vesicles 0.1–1.0 µm in diameter that are released from various cells during cell activation and apoptosis. Depending on the cell of origin, EVs incorporate nuclear, cytoplasmic, and membrane molecules as they detach from the cells, and they express cellular antigens on their surface (10). EVs can be a source of autoantigens and can induce the formation of immune complexes or may be involved in the transfer of miRNA and inflammatory cytokines (11, 12). EVs also have important procoagulant properties based on the availability of phosphatidylserine (PS) exposed on the surface after stimulation (13).

A pivotal study revealed increased levels of platelet-derived EVs (PEVs) in plasma from RA patients, and these levels correlated with disease activity (14), while Boilard et al. demonstrated the presence of a large number of PEVs in the synovial fluid of RA patients (15). This might be a surprising finding because the origin of PEVs in the joint spaces is not known, thus suggesting platelet activation in the joint or the transit of PEVs. Stimulation of the collagen receptor glycoprotein VI on platelets seems to be the key trigger for the collagen-induced generation of PEVs in arthritis pathophysiology (15). However, some data suggest that EVs in the joint can originate from granulocytes or monocytes (16).

Higher proportions of leucocyte-derived EVs (LEVs) have been found in the plasma of RA patients compared to the plasma of patients with osteoarthritis (OA) and healthy controls (HCs), while increased levels of LEVs and monocyte and T-cell–derived EVs were also present in the synovial fluid of patients with RA compared to OA patients (12). Biro E et al. considered the role of complement in the pathogenesis of RA and detected LEVs expressing the complement components C1q, C4, and/or C3 in the synovial fluid of RA patients, but plasma levels of LEVs expressing complement were much lower in both patients with RA and in controls (17). Elevated levels of EVs expressing C1q were found in patients with early RA and remained increased even during the combination therapy with prednisolone, methotrexate, and sulfasalazine and despite improved disease activity as measured by DAS28 scores (18). Additionally, increased EV amounts correlated with the presence of traditional cardiovascular risk factors (diabetes mellitus, hypertension, dyslipidemia and obesity) in a cohort of 114 RA patients, and EVs isolated from RA patients were able to promote endothelial activation *in vitro* (19).

Still, the procoagulant effect of EVs has not previously been explored in patients with RA. This is of particular interest in patients with long-lasting disease and accumulated inflammatory burden leading to the activation of coagulation, diminished fibrinolysis, and accelerated prothrombotic condition. Considering the above observations, we aimed to identify different subpopulations of EVs in the plasma of female patients with established RA in relation to the hemostatic disturbances associated with chronic systemic inflammation in this disease.

## Material And Methods

### Study Participants

Twenty women with established RA referred to the outpatient clinic of the Department of Rheumatology, Clinical Centre Kragujevac, Serbia, were included in the study (mean age 51.85 ± 9.43 years). These patients were previously included as part of a larger study investigating hemostatic disturbances in women with RA (20). RA was diagnosed according to the classification criteria of the American College of Rheumatology (ACR)/European League Against Rheumatism (EULAR) 2010 (21). The exclusion criteria comprised a history of diabetes mellitus, malignancy, or inflammatory bowel disease, liver or renal insufficiency, previous cerebrovascular or cardiovascular disorders (including inherited thrombophilia, antiphospholipid syndrome, and hyperhomocysteinemia), or venous thromboembolism.

RA assessments included a detailed medical history, the presence of extra-articular disease, current disease activity assessed by the 28-point disease activity score (DAS28) (22), and physical function using the Health Assessment Questionnaire (HAQ) (23). All current medications were recorded. Additionally, at the time of blood sampling the patients did not receive any anticoagulants, antithrombotic agents (acetylsalicylic acid), or non-steroidal anti-inflammatory drugs.

Twenty age and sex-matched healthy women (mean age 52.55 ± 7.27 years) were included in the study as healthy controls (HC). None of the women included in the study were taking oral contraceptives or receiving hormone replacement treatment.

Written informed consent was obtained from all participants, and the study protocol was approved by the Ethics Committee of the Clinical Center Kragujevac prior to the onset of the study. The investigation was conducted in accordance with the principles outlined in the Declaration of Helsinki and principles of Good Clinical Practice.

### Blood Sampling

Blood was collected in tubes containing 0.129 M sodium citrate (BD Vacutainer Blood Collection System) using 21-gauge needles (BD Vacutainer needles). Platelet-poor plasma was obtained within 60 min of sampling by centrifugation at 2,000 × *g* for 15 min at room temperature, then divided into aliquots and stored frozen at –70°C until further analysis.

### Isolation of EVs

Platelet-poor plasma was thawed in a water bath for approximately 5 min (37°C) and thereafter centrifuged at 2,000 × *g* for 20 min at room temperature. The supernatant was then re-centrifuged at 13,000 g for 2 min at room temperature. The obtained supernatant was used for further analysis.

### Flow Cytometry Analyses of EVs

Subsequently, 20 µl of the supernatant was incubated for 20 min in the dark with lactadherin-FITC (BD Biosciences, USA) together with CD42a-PE (PEVs; BD Biosciences, USA), CD14-PE [Monocyte EVs (MoEVs); BD Biosciences, USA], CD45-APC [Leucocyte EVs (LEVs); BD Biosciences, USA), CD62P-PE (P-selectin, BD Biosciences, USA), CD62E-PE (Endothelial EVs – E-selectine; BD Biosciences, USA); CD142-PE (Tissue factor (TF), BD Biosciences, USA), CD144-PE (Endothelial EVs; BD Biosciences, USA).

EVs were measured by flow cytometry on a BD FACSCanto™ instrument. The MV-gate was determined using Megamix plus beads FSC (BioCytex, Marseille, France), which is a mix of beads with diameters of 0.1, 0.3, 0.5, and 0.1 µm. EVs were defined as vesicles <1.0 µm in size and positive for lactadherin. Conjugate isotype-matched immunoglobulins (IgG1-FITC, IgG1-PE, and IgG1-APC) with no reactivity against human antigens were used as negative controls to define the positive and negative gates. The absolute number of EVs was calculated by means of the following formula: Concentration (μl) = (Events reading × 550)/(44 × 20), where 550 μl is the total volume of sample in the tube analyzed, 44 μl is the volume of the analyzed sample over 90 s, and 20 μl is the volume of the plasma sample added in the tube (24). The concentration of EVs is expressed as 10^6^ EVs/l, and the intra and interassay coefficients of variation for EV measurements were less than 9%.

### Global Hemostatic Assays

#### Determination of Overall Hemostatic Potential

We employed a modification of the assay described by He et al. (25) in order to assess overall hemostatic potential (OHP) in plasma. Absorbance (Abs) at 405 nm was measured every 12 seconds for 60 minutes, and the area under the curve was calculated by summation of the Abs values (Abs-sum) and expressed as the OHP value. Two additional parameters were also analyzed – the overall coagulation potential (OCP), determined as the area under the fibrin aggregation curve obtained without the addition of tissue plasminogen activator (t-PA), and the overall fibrinolysis potential (OFP), calculated as the difference between the two areas as OFP (%) = ((OCP − OHP)/OCP) × 100. The intra- and inter-assay coefficients of variation for OHP were 1.6% and 6.8% and for OCP were 1.2% and 5.7%, respectively. The detailed protocol was previously described (20).

### Determination of Clot Lysis Time

CLT was determined based on the fibrin aggregation curve for the determination of OHP and was defined as the time from the midpoint of the clear-to-maximum-turbid transition (which corresponds to the “clotting time”) to the midpoint of the maximum turbid-to-clear transition.

### Analysis of Fibrin Clot Formation

Fibrin clot density was assessed with the turbidimetric clotting assay according to the method described by Carter et al. (26). The turbidimetric curve for determination of OCP was used to assess the maximum absorbance (Max Abs) as a measure of the clot density and defined as the increase in Abs from baseline to the maximum value (20).

### Scanning Electron Microscopy

The clots formed during fibrin generation for the determination of OCP were washed with PBS (phosphate-buffered saline) solution and fixed in 2.5% glutaraldehyde in Hepes-buffered saline for 60 min at room temperature and stored at 4°C. Samples were analyzed under an Ultra 55 field emission scanning electron microscope (Carl Zeiss, Oberkochen, Germany) at 3 kV. The detailed protocol was previously described (20).

### Routine Laboratory Analysis

Laboratory analyses of C-reactive protein (CRP) (turbidimetric method, Beckman Coulter AU680 analyser), erythrocyte sedimentation rate (ESR) (Westergren method, Vacuette ESR analyser), fibrinogen concentration (Clauss method, ACL TOP analyser by Instrumentation Laboratory), lipid profile (cholesterol, triglycerides, HDL, and LDL, all by Beckman Coulter AU680 analyser), rheumatoid factor (turbidimetric method, Beckman Coulter AU680 analyser), and ACPA (Roche electrochemiluminescence immunoassay, Cobas e411 analyser), were performed at the Central Laboratory of the Clinical Center Kragujevac.

### Statistical Analysis

All data were analyzed using SPSS 20.0 (IBM Corp. Released 2011), GraphPad Prism 5 (Version for Windows, GraphPad Software, La Jolla California, USA), and FlowJo software 8.7.1 (Treestar, Ashland, OR). The results are expressed as means (SD) or median (IQR) depending on the data type and distribution. Distribution of the data was checked by the Shapiro–Wilk test. Independent samples t-tests (parametric) and Mann–Whitney U-tests (non-parametric) were used to assess the differences in estimated variables between groups. A p-value <0.05 was regarded as statistically significant. Correlation between variables was examined using Spearman correlation analysis.

## Results

### Characteristics of the Study Population

The mean disease duration in patients was 13.0 ± 6.6 years, and disease activity was medium to high (DAS28 was 4.1 ± 1.2) at the moment of blood sampling. The mean HAQ value was 1.2 ± 0.2, and the majority of patients were rheumatoid factor positive (n = 18; 90%) and ACPA positive (n = 20; 100%). All patients were treated with methotrexate (15–25 mg per week) and prednisolone (≤10 mg per day), and only two patients (10%) were in remission (DAS28<2.5). Patients were not previously treated with biologic agents. Three patients had extra-articular manifestations in the form of rheumatoid nodules on the elbows and the small joints of the hands.

There were no differences between the HC and RA patients regarding the presence of traditional CVD risk factors or regarding lipid profile.

Patients with RA had higher CRP, ESR and fibrinogen levels compared to HC. Demographic and clinical characteristics of the participants as well as the presence of traditional CVD risk factors and laboratory parameters are presented in **Table 1**.

**Table 1:** Clinical characteristics and laboratory parameters of the study population.

Subjects characteristics	Healthy controls	RA patients	p value
Number of patients	20	20	
Average age (years)	52.5 ± 7.2	51.8 ± 9.4	NS
BMI (kg/m^2^)	25.3 ± 3.5	25.6 ± 5.2	NS
Disease duration (years)	NA	13.0 ± 6.6	/
DAS28	NA	4.1± 1.2	/
HAQ	NA	1.2 ± 0.2	/
RF positive, n (%)	NA	18 (90)	/
ACPA positive, n (%)	NA	20 (100)	/
Ongoing treatment			
Prednisolone 10mg, n(%)	NA	12 (60)	
Prednisolone 5mg, n(%)	NA	8 (40)	
Methotrexate 15 mg, n(%)	NA	7 (35)	
Methotrexate 20 mg, n(%)	NA	11 (55)	
Methotrexate 25 mg, n(%)	NA	2 (10)	
Smokers (n,%)			
Current smokers	10 (50)	7 (35)	NS
Non-smokers	8 (40)	12 (60)	NS
Past smokers	2 (10)	1 (5)	NS
Cholesterol (mmol/L)	6.2 ± 1.1	5.8 ± 1.1	NS
Triglycerides (mmol/L)	1.4 ± 0.7	1.3 ± 0.5	NS
HDL (mmol/L)	1.56 ± 0.28	1.44 ± 0.24	NS
LDL (mmol/L)	4.0 ± 1.0	3.8 ± 1.1	NS
ESR (mm/h)	10.7 ± 8.5	29.0 (7.0-56.0)	p < 0.001
CRP (mg/L)	1.9 ± 1.0	6.8 (0.8-56.2)	p < 0.001
Fibrinogen (g/L)	3.1 ± 0.6	3.9 ± 0.5	p < 0.001

The values are expressed as means ± SD or median (IQR). BMI, body mass index; DAS28, Disease Activity Score 28; HAQ, Health Assessment Questionnaire; RF, rheumatoid factor; ACPA, citrullinated protein antigen; HDL, High-density lipoprotein; LDL, Low-density lipoprotein; ESR, Erythrocyte sedimentation rate; CRP, C-reactive protein; NA, not applicable; NS, not significant.

### Global Hemostatic Parameters

As presented in **Table 2**, OCP (Abs-sum) and OHP (Abs-sum) were significantly higher, while OFP (%) was significantly lower in RA patients compared to HC. Parameters of fibrin clotting showed higher Max Abs values and longer CLT in RA patients indicating increased clot turbidity and diminished fibrinolysis in these patients.

**Table 2:** Parameters of global hemostatic assays in the study population.

Parameters	Healthy controls	RA patients	p value
OHP (Abs-sum)	345.0 ± 42.8	390.4 ± 60.2	p = 0.003
OCP (Abs-sum)	126.5 ± 28.1	166.3 ± 49.6	p = 0.009
OFP (%)	63.4 ± 6.6	58.0 ± 8.6	p = 0.032
Max Abs	1.6 ± 0.3	1.8 ± 0.2	p = 0.037
CLT (sec)	21.5 ± 3.7	25.1 ± 4.4	p = 0.008

The values are expressed as means ± SD. OHP, Overall hemostasis potential; OCP, Overall coagulation potential; OFP, Overall fibrinolytic potential; Max Abs, increase in absorbance from baseline to maximum value; CLT, clot lysis time.

### EVs

**Figure 1:**
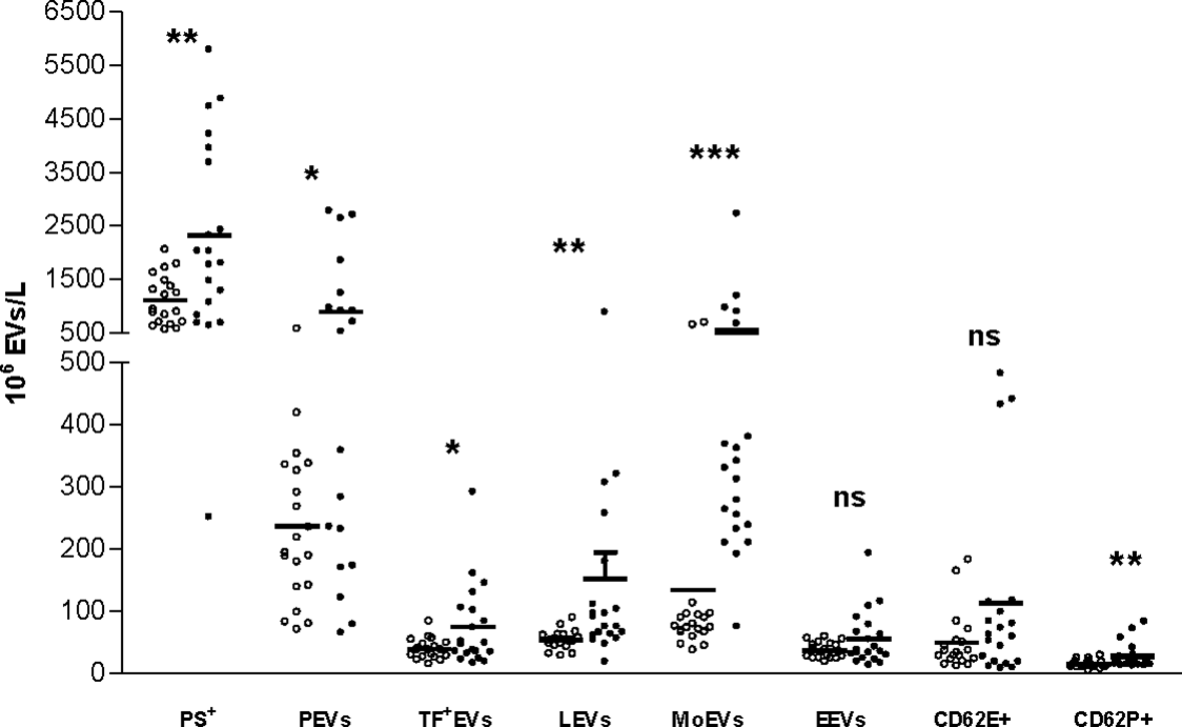
Distribution of different subtypes of EVs in HCs and patients with RA. EVs, extracellular vesicles; Ps, phosphatidylserine; TF, tissue factor; PEVs, platelet-derived EVs; LEVs, leucocyte-derived EVs; MoEVs, monocyte-derived EVs; EEVs, endothelial EVs. *p < 0.05; **p < 0.01; ***p < 0.001; ns, non-significant; white: healthy controls; black: rheumatoid arthritis.

As presented in **Table 3** and **Figure 1**, the concentration of PS^+^ EVs was significantly higher in RA patients compared to HC. Furthermore, PEVs, LEVs, MoEVs and EVs expressing TF and P-selectin were significantly higher in RA patients. EVs of endothelial origin (CD144^+^ or CD62E^+^) were higher in RA patients, but the difference was not statistically significant. The stratified analyses according to the daily corticosteroid dosage have not shown significant differences in the levels of investigated EVs.

**Table 3:** The concentration of different subtypes of EVs in healthy controls and rheumatoid arthritis patients.

EV concentration (10^6^ EVs/l)	Healthy controls (n = 20)	Rheumatoid arthritis patients (n = 20)	p-value
PS^+^ EVs	934.4 (695.6–1479.0)	1918.0 (950.0.5–3822.0)	p = 0.01
PEVs (CD42a^+^)	218.1 (141.3–335.0)	620.9 (202.2–1111.0)	p = 0.02
TF^+^ EVs (CD142^+^)	37.5 (26.2–48.7)	50.3 (35.0–117.8)	p = 0.03
LEVs (CD45^+^)	53.4 (43.4–62.2)	83.1 (62.2–145.3)	p = 0.008
MoEVs (CD14^+^)	74.7 (66.2–94.7)	320.8 (235.0–527.5)	p < 0.001
P-selectin (CD62P^+^)	12.0 (10.0–19.4)	20.0 (14.3–28.7)	p = 0.008
EEVs (CD144^+^)	35.0 (25.0–47.5)	36.2 (26.8–72.2)	NS
E-selectin (CD62E^+^)	33.1 (22.5–52.5)	60.3 (18.7–106.6)	NS

The results are expressed as median (IQR). NS, non-significant difference between the groups; EVs, extracellular vesicles; PS, phosphatidylserine; PEVs, platelet-derived EVs; LEVs, leucocyte-derived EVs; MoEVs, monocyte-derived EVs; EEVs, endothelial EVs.

### Representative Samples

**Figure 2:**
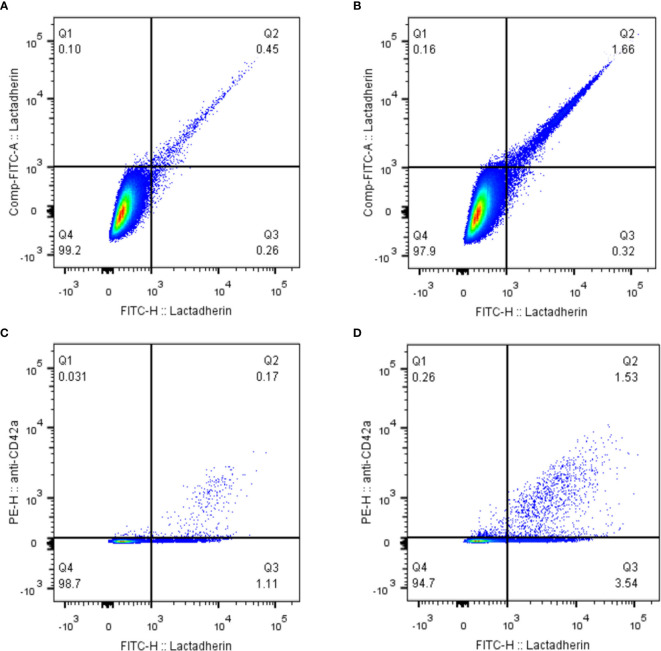
Representative samples of PS+EVs and PEVs. EVs positive for lactadherin (PS^+^EVs) in a control sample **(A)** and RA patient **(B)**. Platelet-derived EVs in a control sample **(C)** and RA patient **(D)**. EVs, extracellular vesicles.

Representative dot-plots for PS^+^EVs (Lactadherin+ EVs) regardless of cellular origin, together with PEVs (CD42a+) as the most common EVs, are presented on **Figure 2**.

### Correlations

The correlations between the levels EVs and inflammatory parameters ESR and CRP as well as the parameters of the global hemostatic assays are presented in **Table 4**.

**Table 4:** Correlations between the levels of EVs and inflammatory parameters and the parameters of the global haemostatic assays.

Parameters	CRP	ESR	OCP	OHP	OFP	Max Abs	CLT
PS^+^EVs	p = 0.048 *r = 0.32*	p = 0.009 *r = 0.41*	p = 0.013 *r = 0.39*	p = 0.001 *r = 0.51*	p = 0.006 *r = –0.43*	p = 0.001 *r = 0.511*	p = 0.002 *r = 0.467*
PEVs (CD42a^+^)	p = 0.031 *r = 0.34*	p = 0.007 *r = 0.42*	p = 0.039 *r = 0.33*	p = 0.002 *r = 0.47*	p = 0.006 *r = –0.43*	p = 0.004 *r = 0.441*	p = 0.006 *r = 0.426*
TF^+^EVs (CD142^+^)	p > 0.05	p > 0.05	p > 0.05	p > 0.05	p > 0.05	p > 0.05	p > 0.05
LEVs (CD45^+^)	p > 0.05	p > 0.05	p > 0.05	p > 0.05	p > 0.05	p = 0.024 *r = 0.355*	p = 0.049 *r = 0.313*
MoEVs (CD14^+^)	p = 0.001 *r = 0.51*	p = 0.005 *r = 0.44*	p > 0.05	p > 0.05	p = 0.031 *r = –0.34*	p = 0.002 *r = 0.484*	p = 0.005 *r = 0.439*
P-selectin (CD62P^+^)	p = 0.015 *r = 0.38*	p = 0.004 *r = 0.47*	p > 0.05	p = 0.014 *r = 0.39*	p = 0.015 *r = –0.38*	p > 0.05	p = 0.007 *r = 0.420*
EEVs (CD144^+^)	p > 0.05	p > 0.05	p > 0.05	p > 0.05	p > 0.05	p > 0.05	p > 0.05
E-selectin (CD62E^+^)	p > 0.05	p > 0.05	p > 0.05	p > 0.05	p = 0.041 *r = –0.32*	p > 0.05	p = 0.028 *r = 0.349*

EVs, extracellular vesicles; PS, phosphatidylserine; TF, tissue factor; PEVs, platelet-derived EVs; LEVs, leucocyte-derived EVs; MoEVs, monocyte-derived EVs; EEVs, endothelial EVs; OHP, Overall hemostasis potential; OCP, Overall coagulation potential; OFP, Overall fibrinolytic potential; Max Abs, increase in absorbance from baseline to maximum value.

### Analysis of Clot Structure

**Figure 3:**
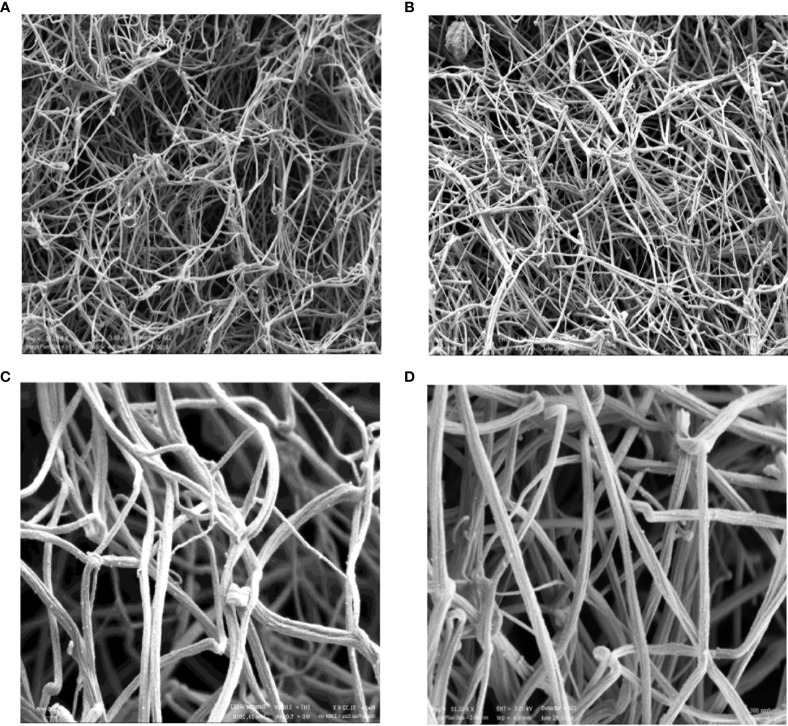
Scanning electron microscopic images of representative fibrin clots. HC **(A, C)** and RA patient **(B, D)**. The magnification 1 μm was used for **(A, B)** and 300 nm for **(C, D)**. HC, healthy control.

Scanning electron microscopy showed dense fibrin structures in the plasma from an RA patient compared to the control sample. The clot from the RA patient was made of thinner fibers that were tightly packed into the network with smaller intrinsic pores and thus less susceptible to fibrinolysis (**Figure 3**), and this was similar to our previous findings of fibrin clots in women with RA in regard to the menopausal status (20).

Measurement of the fiber thickness of the representative samples was performed in five different areas, and the mean value of 10 fibers/area was used as the final measure of fiber thickness in these samples. Thinner fibers were found in the patient compared with control (136 nm ± 49 nm vs 157.0 ± 56.0 nm).

## Discussion

We determined the presence of circulating EVs in relation to hemostatic disturbances in women with established RA, indicating the presence of a hypercoagulable state in these patients. Despite treatment, the patients with RA included in our study presented with ongoing inflammatory activity contributing to the extensive production of circulating EVs of platelet, leucocyte, and monocyte origin as well as increased expression of PS, TF, and P-selectin on the surface of EVs. In addition, amplified fibrin formation together with increased clot turbidity and diminished fibrinolysis was confirmed in these patients as measured by the global hemostatic parameters OCP, OHP, Max Abs, and OFP, respectively (20). We postulate that EVs play an important role in the delicate interaction between inflammatory processes and the activation of coagulation in the course of RA. The major advantage of our study is the investigation of circulating EVs of different origin in relation to hemostatic and inflammatory parameters in a homogenous group of RA patients in comparison to strictly matched control subjects.

While the formation of EVs is a physiological response to cell activation or cell death, a dramatic increase in circulating EVs has been detected in conditions associated with inflammatory states of different severity and different prothrombotic conditions (27–29). Identification of EV origin is vital because it indicates the dominant cell types involved in the pathogenesis of these disorders (14, 18, 30, 31). Several studies have tried to elucidate the role of circulating EVs as well as EVs detected in the synovial fluid in the pathogenesis of RA, as reviewed in Boilard et al. (32).

We have shown elevated levels of EVs expressing PS irrespective of the cell origin in plasma from patients with RA. The phospholipid membranes of EVs containing PS represent an adequate platform for the activation of coagulation processes leading to increased thrombin and fibrin formation, as measured by OCP and OHP (28, 29). Further, we present the association between the intensity of inflammation in RA and the concentration of released PS^+^EVs. Thus, the negatively charged phospholipid membrane of EVs might play an important role in the interactions between the inflammatory state and procoagulant condition in the circulation of RA patients.

In line with previous studies, our results verify that the majority of circulating EVs in RA patients are of platelet origin (14, 29, 33), reflecting platelet activation in RA as an essential part of the inflammatory reaction. PEVs are involved in the interactions between platelets and inflammatory cells and in production of pro-inflammatory cytokines (31). Previously, a direct correlation was found between the levels of circulating PEVs and disease activity in RA patients (14, 29, 33). We were not able to confirm this association, probably due to the small number of investigated patients. Still, there was clear association of circulating PEVs with the inflammatory parameters, as well as the parameters of activation of coagulation and impaired fibrinolysis in our patients. As an additional marker of platelet activation, we noticed increased expression of P-selectin on circulating EVs in plasma from RA patients. P-selectin mediates the interaction between platelets and neutrophils (11) and may be a sign of ongoing platelet activation in RA, even if the disease is clinically silent (34). Amplified expression of P-selectin was also found in the synovial fluid of RA patients, though not as high as in the circulation (33).

Moreover, circulating PEVs have the ability to incorporate into the fibrin clots (35). We have recently confirmed the close interaction between PEVs and fibrin fibers, particularly at branch points and junctions (24). This finding may suggest intensified fibrin formation around the surface of PEVs, following the activation of platelets and the propagation phase of thrombin generation. Denser fibrin clots formed upon extensive platelet activation and augmented expression of PEVs in RA might therefore be revealed by the increased fibrin turbidity as measured by the Max Abs in our study. Scanning electron microscope pictures of denser fibrin composed of thinner fibers and with smaller intrinsic pores in RA patients confirm this finding, while decreased OFP together with prolonged CLT reflects the reduced fibrinolytic capacity as a consequence. Formatin of fibrin clots with a more prothrombotic phenotype in the presence of PEVs may be in agreement with the work of Knijff-Dutmeret et al. suggesting a possible association between PEVs and the development of CVD in patients with RA (14).

Elevated levels of circulating MoEVs were also found in the RA patients. The interactions between platelets and monocytes are well documented, indicating the accelerated generation of TFs by activated monocytes and the particularly important role of PEVs and MoEVs in this process (36–38). Therefore, our finding of increased TF expression on circulating EVs in RA patients is not surprising. Previously, higher proportions of MoEVs were reported in RA patients with high disease activity compared to patients in remission (39), and we confirmed the association with the inflammatory markers in our cohort. We believe that the elevated concentrations of PEVs together with MoEVs and EVs expressing P-selectin and TF could be potential biomarkers for the inflammatory and procoagulant activity in RA.

Apart from circulating EVs, synovial fluid obtained from the joints of RA patients contains EVs derived from monocytes, granulocytes, T-cells, B-cells, and even platelets (12, 15, 16, 40–43). The major disadvantage of our study is the lack of investigation of EVs in the synovial fluid. The majority of our patients presented with medium-high disease activity as measured by DAS-28 score, but a sufficient amount of synovial fluid was not available for sampling during the study period.

Systemic inflammation in RA is accompanied by activation of the vascular endothelium (44). Still, the levels of EVs expressing E-selectin and endothelium-derived EVs were not significantly different in plasma from RA patients compared to HC. E-selectin might only be transiently expressed on endothelial cells in RA patients and therefore might be difficult to detect (34). In a study by Viñuela–Berni et al., the administration of immunosuppressive therapy (methotrexate, sulphasalazine, and low-dose glucocorticoids) led to a significant reduction of the plasma levels of CD62E^+^ EVs (39). Further, Hjeltnes et al. showed that administration of methotrexate or a combination of methotrexate and TNFα inhibitor decreased the serum level of E-selectin (45), and the levels of endothelium-derived EVs levels increased and then decreased after four months of anti-TNFα therapy (46). The expression of endothelium-derived EVs in comparison to PEVs was rather low and might therefore be more sensitive to the ongoing immunosuppressive therapy in the investigated group of patients.

The important limitation of our study is small number of investigated subjects. However, the patients were well characterized for the presence of traditional cardiovascular risk factors and ongoing medications and had moderate to high disease activity, thus enabling an assessment in a real-life setting.

## Conclusion

We demonstrate elevated levels of circulating EVs in patients with established RA in relation to inflammatory burden and coagulation activation. Larger studies are needed to confirm these preliminary findings.

## Data Availability Statement

The raw data supporting the conclusions of this article will be made available by the authors, without undue reservation.

## Ethics Statement

The studies involving human participants were reviewed and approved by Ethics Committee of the Clinical Center Kragujevac, Serbia. The patients/participants provided their written informed consent to participate in this study.

## Author Contributions

AS and MV collected samples. AS, IP, and YZ performed the experiments. AS and AA performed statistical analyses. AA and VJ designed the study. AA, VJ, and MV supervised the manuscript. All authors contributed to the article and approved the submitted version.

## Funding

The study was performed by grants provided by Region Stockholm (ALF project), King Gustaf the V-80 years foundation and the Swedish Rheumatism Association.

## Conflict of Interest

The authors declare that the research was conducted in the absence of any commercial or financial relationships that could be construed as a potential conflict of interest.

## Publisher’s Note

All claims expressed in this article are solely those of the authors and do not necessarily represent those of their affiliated organizations, or those of the publisher, the editors and the reviewers. Any product that may be evaluated in this article, or claim that may be made by its manufacturer, is not guaranteed or endorsed by the publisher.
